# Production of scandium radionuclides for theranostic applications: towards standardization of quality requirements

**DOI:** 10.1186/s41181-021-00131-2

**Published:** 2021-05-25

**Authors:** R. Mikolajczak, S. Huclier-Markai, C. Alliot, F. Haddad, D. Szikra, V. Forgacs, P. Garnuszek

**Affiliations:** 1 Radioisotope Centre POLATOM, National Centre for Nuclear Research, Andrzej Soltan 7, 05-400 Otwock, Poland; 2 Laboratoire Subatech, UMR 6457, IMT Nantes Atlantique /CNRS-IN2P3 / Université de Nantes, 4 Rue A. Kastler, BP 20722, 44307 Nantes Cedex 3, France; 3 ARRONAX GIP, 1 rue Aronnax, 44817 Nantes Cedex, France; 4 CRCINA, Inserm / CNRS / Université de Nantes, 8 quai Moncousu, 44007 Nantes Cedex 1, France; 5 Faculty of Medicine, Department of Medical Imaging, Division of Nuclear Medicine and Translational Imaging, University of Debrecen, Nagyerdei krt. 98, Debrecen, 4032 Hungary; 6 Scanomed Ltd., Nagyerdei krt. 98, Debrecen, 4032 Hungary

**Keywords:** Scandium radionuclides, Accelerator- and nuclear reactor production, Coordination, Radiolabeling, In vivo studies, Quality specifications

## Abstract

In the frame of “precision medicine”, the scandium radionuclides have recently received considerable interest, providing personalised adjustment of radiation characteristics to optimize the efficiency of medical care or therapeutic benefit for particular groups of patients. Radionuclides of scandium, namely scandium-43 and scandium-44 (^43/44^Sc) as positron emitters and scandium-47 (^47^Sc), beta-radiation emitter, seem to fit ideally into the concept of theranostic pair. This paper aims to review the work on scandium isotopes production, coordination chemistry, radiolabeling, preclinical studies and the very first clinical studies. Finally, standardized procedures for scandium-based radiopharmaceuticals have been proposed as a basis to pave the way for elaboration of the Ph.Eur. monographs for perspective scandium radionuclides.

## Introduction

In the quest for new radionuclides providing personalised adjustment of radiation characteristics to optimize the efficiency of medical care or therapeutic benefit for particular groups of patients (the so called “precision medicine”), the scandium radionuclides have recently received considerable interest. In the last two decades, several new radionuclides for diagnostic imaging and therapy have been successfully introduced to clinical practice. Additionally, using the same targeting vectors and the combination of positron emitter gallium-68 (^68^Ga) for diagnostic imaging and matching therapeutic counterpart beta-emitting radionuclides such as lutetium-177 (^177^Lu) and yttrium-90 (^90^Y) or, recently, alpha emitters bismuth-213 (^213^Bi) and actinium-225 (^225^Ac), for therapeutic use, has been recognized as a clear advantage over currently available treatment options. From this perspective, radionuclides of scandium, namely scandium-43 and scandium-44 (^43/44^Sc) as positron emitters and scandium-47 (^47^Sc), beta-radiation emitter, seem to fit ideally into the concept of theranostic pair (Mausner and Srivastava 1993; Huclier-Markai et al. 2018). Scandium-47 is also a low energy γ-emitter and allows SPECT and planar imaging. Therapeutic potential of ^47^Sc was studied at the Brookhaven National Laboratory already in the 1990s (Mausner and Srivastava 1993; Pietrelli et al. 1992). However, the rapid growth of the scandium radionuclides applications started only after the introduction of ^68^Ga-labelled compounds for PET diagnosis in the early 2000s. Scandium-44 was proposed as a potential alternative to ^68^Ga for clinical applications in 2010 by the group of Rösch in Mainz, Germany (Roesch 2012; Pruszynski et al. 2010). Similarities in chemistry between ^68^Ga and ^44^Sc and their different physical properties have opened a wider avenue for applications of other Sc radionuclides.

Scandium radioisotopes can be produced in accelerators and in nuclear reactors (Huclier-Markai et al. 2018; Muller et al. 2018; Mikolajczak et al. 2019). In particular, due to their large number, biomedical cyclotrons accelerating protons up to 20 MeV are expected to provide ^43^Sc and ^44g^Sc in quantities that would allow their wider use in diagnostic imaging (Roesch 2012; Synowiecki et al. 2018). Similarly, cyclotrons accelerating protons to higher energies, nuclear reactors and electron linear accelerators (linacs) might be a source of ^47^Sc for therapy (Domnanich et al. 2017a; Jalilian et al. 2020; Qaim 2019). However, due to several production routes possible, the unification of quality parameters of Sc radionuclides has not been yet attempted.

## Scandium isotopes with medical potential

Natural scandium has only one stable isotope, scandium-45 (^45^Sc). Several other scandium isotopes can be produced artificially, however, most of them are short-lived, in seconds range (http://kcvs.ca/isotopesmatter/iupacMaterials/javascript/Interactive%20Periodic%20Table%20of%20the%20Isotopes/HTML5/pdf-elements/scandium.pdf). Those with atomic mass smaller than 45 decay by emission of positrons, while those with atomic mass greater than 45 emit electrons. Characteristics of Sc radioisotopes with longer half-lives are given in Table 1 (adapted from Pawlak et al. 2019). Among them, ^43^Sc, ^44,44m^Sc and ^47^Sc are suitable for medical applications.

**Table 1:** Physical characteristics of relevant Sc radioisotopes

Radionuclide	Half-life	Decay mode	Energy of particles or photons (keV)
^43^Sc	3.89 h	β^+^	1198
			825
		γ	372
^44g^Sc	3.97 h^a^	β^+^	1475
		γ	1157
^44m^Sc	58.6 h	γ	271
			1002
			1126
			1157
^46^Sc	83.79 d	β^−^	357
		γ	889
			1121
^47^Sc	3.35 d	β^−^	600
			439
		γ	153
			1120
^48^Sc	43.7 h	β^−^	654
			485
		γ	183
			1037
			1312

^a^It is worth noting that most of the sources provide the half-life of ^44^Sc T_1/2_ = 3.97 h. This value has been recently re-determined by Garcia-Tarrano et al. (Garcia-Torano et al. 2016) to be T_1/2_ = 4.042 h, about 2% higher than the earlier recommended value

## Nuclear data and production methodologies

Production routes for ^43^Sc, ^44g, 44m^Sc and ^47^Sc use either calcium, titanium or vanadium targets, typically enriched for the desired isotope. A summary of nuclear reactions, decay data, cross-sections, and targetry is given in Table 2, together with the chemical form, purity, enrichment and cost assessment of the target material (where available). For each of the radionuclides, we also report the production mode (i.e. accelerator, nuclear reactor, radionuclide generator) and examples of production facilities that have already implemented their production.

**Table 2:** Nuclear Data (NUDAT2), production methodologies and irradiation sites of scandium radionuclides

Isotope	Decay data	Nuclear reaction	Cross section (Max value)	Beam energy (MeV)	Enrichment of target material	Composition/ chemical form	Cost +++ = expensive --- = not expensive	Irradiation site / type
Sc-43	T_1/2_ = 3.89 h <E_β_^+^ > = 476 keV (88.1%) Eγ =372 keV (22.5%)	^43^Ca(p,n)^43^Sc	300 mb	6–13	natural abundance 0.135% Max enrichment 90%	^nat^CaCO_3_ ^43^CaCO_3_	++	PSI (S) (Van der Muelen 2015)
		^44^Ca(p,2n)^43^Sc	~ 170 mb	18–27	natural abundance of only 2.09% Max enrichment 99%	^44^CaCO_3_	++	
		^46^Ti(p,α)^43^Sc Krajewski et al., 2012	45 mb	11–21	natural abundance of 8.25% Max enrichment 97%	^46^TiO_2_	+	PSI (S) University of Alabama at Birmingham (USA)
		^42^Ca(d,n)^43^Sc	200 mb	2–11	natural abundance of only 0.647% Max enrichment 96.8%	^42^Ca ^42^CaCO_3_	+++	
		^nat^Ca(α,n)^43^Ti (T_1/2_ = 509 ms)➔^43^Sc (Koning, 2016; Howard, 1974) ^nat^Ca(α,p)^43^Sc (Synowiecki et al. 2018; Domnanich et al. 2017a)	570 mb (Howard, 1974)	10–19	natural abundance 96.94% (as ^40^Ca) no need for enrichment	^40^Ca (not so easy to handle) ^nat^CaCO_3_ ^40^CaCO_3_	++	cyclotron with alpha beam HIL, Warsaw (PL)
Sc-44	T_1/2_ = 3.97 h <E_β_^+^ > = 632 keV (94.27%) Eγ = 1157 keV (99.9%)	^45^Sc(p,2n)^44^Ti (T_1/2_ = 60y) (generator ^44^Ti➔^44^Sc)	45 mb	17–31	Natural abundance	^45^Sc	+++	LANL + BNL (USA)
		^nat^Ca(p,n)^44^Sc	10 mb	7–15	natural abundance 96.94% (as ^40^Ca) no need for enrichment	^nat^Ca(NO_3_)_2_, 4H_2_0	–	(http://kcvs.ca/isotopesmatter/iupacMaterials/javascript/Interactive%20Periodic%20Table%20of%20the%20Isotopes/HTML5/pdf-elements/scandium.pdf) Local use Univ. Wisconsin (USA) / cyclotron Triumf (CA)
		^44^Ca(p,n)^44^Sc/^44m^Sc	700 mb	7–15	natural abundance of only 2.09% Max enrichment 99%	^44^CaCO_3_	++	PSI (S) Univ. Alabama Birmingham (USA)
		^44^Ca(p,n)^44^Sc/^44m^Sc	700 mb	7–15	Max enrichment 99%	^44^CaO	++	PSI (S) (van der Meulen et al. 2020)
		^44^Ca(d,2 n) ^44^Sc/^44m^Sc	540 mb	11–25	natural abundance of only 2.09% Max enrichment 99%	^44^CaCO_3_	++	Arronax (F)
		^47^Ti (p,α)^44^Sc	70 mb	12–20	natural abundance 7.44% Max enrichment > 95%	^47^TiO_2_	+	
^**47**^**Sc**	T_1/2_ = 3.349 d <E_β-_ > = 162 keV(100%) Eγ = 159 keV (68.3%)	^47^Ti(n,p)^47^Sc	~250mb	Fast neutron	natural abundance 7.44% Max enrichment > 95%	^47^TiO_2_	+	nuclear reactor (Walczak et al. 2015; Szkliniarz et al. 2016; Minegishi et al. 2016; Carzaniga et al. 2019)
		^46^Ca(n,γ)^47^Ca → ^47^Sc	0.74 b	Thermal neutron	natural abundance of only 0.004% Max enrichment 24.8%	^46^CaCO_3_	+++	nuclear reactor: ILL (F) (Minegishi et al. 2016) MARIA(Pl) (Carzaniga and Braccini 2019) ETRR-2 (ET) (Sitarz et al. 2018) Dhruva (IND) (Filosofov et al. 2010)
	Direct reaction on Ti targets	^50^Ti(p,α)^47^Sc^a^	~ 25 mb	15–30	natural abundance 5.18% Max enrichment 83%	^50^TiO_2_	+++	University of Alabama at Birmingham (USA)
		^48^Ti(p,2p)^47^Sc	~ 30 mb	30–100	natural abundance 73.72% Max enrichment > 96%	^48^TiO_2_	–	high energy accelerators, BNL and LANL (USA)
		^50^Ti(d,αn)^47^Sc	> 60mb		natural abundance 5.18% Max enrichment 83%	^50^TiO_2_	+++	
		^49^Ti(d,α)^47^Sc	~ 40 mb		natural abundance 5.41% max enrichment 92.4	^49^TiO_2_	++	
		^47^Ti(d,2p)^47^Sc	~ 40 mb		natural abundance 7.44% Max enrichment > 95%	^47^TiO_2_	+	
	Direct reaction on Ca targets	^48^Ca(p,2n)^47^Sc^,^	~ 800 mb	12–26	natural abundance 0.187% Max enrichment 97.1%	^48^CaCO_3_	++	(Krajewski et al. 2013; Domnanich et al. 2017b)
		^44^Ca(α,p) ^47^Sc	~ 120 mb	10–20	natural abundance 2.09% Max enrichment 99%	^44^CaO	++	(Domnanich et al. 2017a)
	Direct reaction on V targets	^51^V(p,αp)^47^Sc	~ 15 mb	30–40	natural abundance	^nat^V	–	(van der Meulen et al. 2015)
	Electron Linear Accelerator	^48^Ti(γ,p)^47^Sc	~ 28 mb	16–28	natural abundance 73.72% Max enrichment > 96%	^48^TiO_2_	–	LANL (USA)

^a^E Gadiooli et al., Z. Phys A Atoms and Nucl D4060001, 39, 301, 289-300, 1981

### ^43^Sc production

$$ {}^{43}\mathrm{Sc}\ \Big({\mathrm{T}}_{1/2}=3.89\;\mathrm{h},{{\mathrm{E}}_{\upbeta}}^{+}=476\;\mathrm{keV}\ \left(88.1\%\right),\mathrm{branching}\ \mathrm{ratio}\ {\upbeta}^{+}:88\%,\mathrm{E}\upgamma =372\;\mathrm{keV}\ \left(22.5\%\right) $$

Several production routes are possible for ^43^Sc, involving proton, deuteron or alpha beams (Chaple and Lapi 2018; Braccini 2016). These differents production routes are described in more detail below.

#### Production of ^43^Sc using alpha beams

Reasonable activities of ^43^Sc can be produced by irradiating a ^nat^Ca target. It is obtained both directly throught ^40^Ca(α,p)^43^Sc and ^40^Ca(α,n)^43^Ti (T_1/2_ = 509 ms) → ^43^Sc nuclear reactions, using accelerators. Effective production of ^43^Sc using alpha beam was presented by Walczak et al. (Walczak et al. 2015). Szkliniarz et al. (Szkliniarz et al. 2016) irradiated natural calcium as calcium carbonate with 20 MeV alpha particle beam. Resulting ^43^Ti decays to ^43^Sc during the irradiation and no chemistry is needed to separate ^43^Sc from ^43^Ti due to its very short half-life. Impurities ^44m^Sc and ^44g^Sc are formed due to the ^42^Ca content (0.65%) in the natural calcium target as well as ^46^Sc and ^47^Sc due to the ^44^Ca content (2% of natural calcium). The ^42^Ca(α,np + pn)^46^Sc nuclear reaction has a maximum cross-section around 30 MeV, thus its formation can be decreased by using lower beam energy. Conversely, ^47^Sc formation cannot be avoided, as it has a similar maximum (17 MeV) as the ^40^Ca(α,p)^43^Sc reaction (14 MeV). The radionuclidic purity of the produced ^43^Sc was 99.95% at the end of bombardment (EOB) and 98.9% at 20 h after EOB. The radionuclidic purity decreases with time as ^43^Sc half life is lower than that of ^44m^Sc, ^46^Sc and ^47^Sc. The use of enriched ^40^CaCO_3_ target material decreased dramatically the level of radionuclidic impurities to 1.5 × 10^− 5^%. Thick target yield for irradiation of calcium metal was found to be 240 MBq/μAh, from which 15 GBq production yield was extrapolated for 4 h irradiation with a 25 μA alpha particle beam. Based on the experimental results, 1 GBq yield of ^44m^Sc is expected for high beam current alpha irradiation (12 h, 25 μA) of enriched ^42^CaCO_3_ target (Szkliniarz et al. 2016). Using alpha beam irradiation, Minegishi et al. (Minegishi et al. 2016) reported a remote method for ^43^Sc production on an unsolidified, powder calcium oxide (CaO) target. The powdery CaO target material was dissolved in situ in HCl in the target box and remotely recovered as a radio-Sc solution as it is done when using liquid targets. The yield of ^43^Sc following isolation via a precipitation method with a typical 0.22 μm sterile filter was 54.8 MBq/μAh at the end of separation (approximately 1.5 h from the EOB).

#### Production of ^43^Sc using protons

The number of cyclotrons providing regular and intense alpha beams is limited, therefore, production methods using medical cyclotrons (proton beams) are gaining more interest. Domnanich et al. (Domnanich et al. 2017b) demonstrated that ^43^Sc can be produced at a medical cyclotron via proton irradiation of enriched ^43^Ca or ^46^Ti oxide target. The production via the ^46^Ti(p,α)^43^Sc nuclear reaction yielded a ^43^Sc activity around 200 MBq and high radionuclidic purity (> 98%) at the end of a 7 h irradiation when using 97% enriched ^46^Ti. The production via the ^43^Ca(p,n)^43^Sc nuclear reaction resulted in higher quantities of ^43^Sc, but the product consisted of a mixture of ^43^Sc and ^44g^Sc and an activity ratio of 2:1 when using 57.9% enriched ^43^CaCO_3_. This may be increased with higher enrichment of the ^43^Ca target (max available is 90% at the moment). However, the question remains whether ^44g^Sc is a real problem as it decays with almost the same half-life by emitting a positron, as ^43^Sc.

#### Production of ^43^Sc using deuterons

^43^Sc can be also produced by deuteron bombardment of an enriched ^42^Ca oxide target, although the method has not been yet practically utilized. Recently, measurements of the ^43^Sc production cross-section in the reaction ^42^Ca(d,n)^43^Sc with a deuteron beam have been reported by Carzaniga et al. (Carzaniga et al. 2019; Carzaniga and Braccini 2019). The authors also studied practical aspects of producing ^43^Sc via this route using commercially available targets. Yet, the limited number of medical cyclotrons currently in operation offering deuteron beams prevents the wider application of this method.

In summary, ^43^Sc can be produced with proton and deuteron beams that are easily available worldwide, the use of enriched (and expensive) target material, ^43^Ca or ^44^Ca, is mandatory due to their low natural abundance. When proton irradiation route is preferred, the issue of co-produced ^44g^Sc in ^43^Sc needs to be addressed (Sitarz et al. 2018). On the other hand, irradiation with an alpha particle beam allows to use directly natural calcium as the nuclear reaction of interest involves ^40^Ca (natural abundance is 96.94%). However, such a kind of beam is only available in a few places in the world. Though, higher radionuclidic purity can be obtained using enriched ^40^Ca target, that is more affordable than other enriched calcium materials.

### ^44g^Sc production

$$ {}^{44\mathrm{g}}\mathrm{Sc}\ \left({\mathrm{T}}_{1/2}=3.97\;\mathrm{h},{{\mathrm{E}}_{\upbeta}}^{+}=632\;\mathrm{keV}\ \left(94.27\%\right),\mathrm{branching}\ \mathrm{ratio}\ {\upbeta}^{+}:94.3\%,{\mathrm{E}}_{\upgamma}=1157\;\mathrm{keV}\ \left(99.9\%\right)\right) $$

^44g^Sc can be obtained directly by irradiation of an enriched ^44^Ca target of different chemical forms or via the use of the ^44^Ti/^44g^Sc generator. The main difference of the two methods is that in the first case, there is a co-production of ^44m^Sc (T_1/2_ = 58.61 h) which is not present in the generator produced ^44^Sc.

#### ^44^Ti/^44g^Sc generators

^44^Ti/^44g^Sc generator has been proposed as a source of ^44g^Sc (Roesch 2012; Filosofov et al. 2010; Radchenko et al. 2016). Although the generator method has been extensively investigated, only a small number of facilities worldwide use these generators. The parent radionuclide, titanium-44 (T_1/2_ = 60 y) is produced through the nuclear reaction ^45^Sc(p,2n)^44^Ti. Production of mCi amount of ^44^Ti is difficult due to its long half-life and the low cross section (probability) of this nuclear reaction. As an example, irradiation of 1.5 g of Sc target material produced about 185 MBq (5 mCi) (Chaple and Lapi 2018).

#### Production of ^44^Sc using protons

It is thus easier to use an accelerator to produce ^44g^Sc. Medical cyclotron can be used for proton irradiation of a target containing either natural (Severin et al. 2012) or ^44^Ca enriched target (Krajewski et al. 2013). Enriched targets are preferred to avoid the production of contaminants such as ^46,48^Sc (Domnanich et al. 2017a). The higher the ^44^Ca enrichment, the higher the final radionuclidic purity. However, during the irradiation, ^44m^Sc is co-produced which may be either an advantage or a disadvantage depending on the application. Indeed, its long half life allows ^44m^Sc (T_1/2_ = 58.6 h) to be used to image slow biological processes. This is possible since ^44m^Sc/^44g^Sc can act as an in-vivo generator (Alliot et al., 2015a). The production of ^44g^Sc via the ^44^Ca(p,n)^44g^Sc nuclear reaction has been implemented at the research cyclotron at Paul Scherrer Institute, Zurich, providing this radionuclide with high radionuclidic purity (> 99%) and at high activities (> 2 GBq) (van der Meulen et al. 2015). This would be the method of choice when one wants to produce cost effectively ^44g^Sc. Other targets or projectiles have also been used, presenting some advantages for dedicated cases: higher contribution of ^44m^Sc for example or lower cost when using ^nat^Ca. The use of natural calcium metal provides cost-efficient access to ^44g^Sc for preclinical experiments. It can be pressed into the cavity of an appropriate target holder (coin or shuttle) and irradiated with high currents, as it has good heat conductivity. However, contaminating radiometals prevent the use of this production route for human application. Exotic production routes have been also studied using ^47^Ti in a ^47^Ti (p,α)^44^Sc nuclear reaction (Loveless et al. 2021).

#### Production of ^44^Sc using deuterons

Deuteron beams can also be used for production of ^44^Sc in a nuclear reaction ^44^Ca(d,2n) ^44^Sc/^44m^Sc (Alliot et al. 2015a). A proof of principle has been demonstrated at low beam current (0.3 μA). An activity of 90 MBq (4 h after EOB) can be obtained with a 3 h bombardment on a 500 μm thick ^44^CaCO_3_ target at 17 MeV (Alliot et al. 2015b). This study showed also that the use of deuterons allows to increase the production of ^44m^Sc with respect to proton irradiation keeping ^44g^Sc at the same level as for protons (Duchemin et al. 2015; Duchemin et al. 2016). However, to favor ^44m^Sc production the use of alpha beam is recommended (Szkliniarz et al. 2016).

### ^47^Sc production

$$ {}^{47}\mathrm{Sc}\ \left({\mathrm{T}}_{1/2}=3.349\ \mathrm{d},{\mathrm{E}}_{\upbeta -}=162\;\mathrm{keV}\ \left(100\%\right),{\mathrm{E}}_{\upgamma}=159\;\mathrm{keV}\ \left(68.3\%\right)\right) $$

Scandium-47 can be produced via several different nuclear reactions using a nuclear reactor, a cyclotron, or a linac (Srivastava 2013).

#### Production of ^47^Sc using neutrons

In a nuclear reactor, practically n.c.a ^47^Sc is produced via ^47^Ti(n,p)^47^Sc nuclear reaction by irradiation of ^47^Ti target with fast neutrons (energy greater than 1 MeV). The ^47^Ti(n,p)^47^Sc route also requires an enriched target, though ^47^Ti oxide is available with very high enrichment and at a reasonable cost. Thus, using the ^47^Ti(n,p)^47^Sc nuclear reaction could provide quantities sufficient for therapy. For example, in a HFIR reactor (Oak Ridge National Laboratory) a 3.35 day (one half-life of ^47^Sc) irradiation of 10 g target could produce approximately 2800 GBq of ^47^Sc at EOB (Kolsky et al. 1998; Mausner et al. 1993). However, along with ^47^Sc, ^46^Sc is co-produced (Domnanich et al. 2017a; Bokhari et al. 2009; Bartoś et al. 2012). This method suffers from the limited number of facilities delivering fast neutrons.

Alternatively, ^47^Sc can be produced with thermal neutrons (Mausner 1998; Deilami-Nezhad et al. 2016) that are more widely available. Neutron capture on ^46^Ca produces ^47^Ca (T_1/2_ = 4.5 d), which decays into ^47^Sc by β^−^ emission: ^46^Ca(n,γ)^47^Ca → ^47^Sc, and the obtained ^47^Ca can be further exploited as the ^47^Ca/^47^Sc generator system (Mausner 1998). This method suffers mainly from the low natural abundance of ^46^Ca (0.004%). Still, ^47^Sc production from neutron irradiated natural Ca target was shown to be feasible (Gizawy et al. 2020). Nontheless, to obtain significant activity of ^47^Ca the target enriched in ^46^Ca must be used (presently ^46^Ca is available with a maximum 30% enrichment) which is rather expensive (Chakravarty et al. 2017) but recycling would allow to drastically reduce the cost. For comparison, when 0,97 mg of ^46^Ca (48.5 mg of 5% enriched [^46^Ca]CaCO_3_) was irradiated in a thermal neutron flux 1.2 × 10^14^ ns^− 1^ cm^− 2^ for 6 days, around 700 MBq of ^47^Ca and 350 MBq of ^47^Sc were produced at EOB (Pawlak et al. 2019). The produced ^47^Ca decays to ^47^Sc with a half-life of 4.5 days, which is longer than the half-life of ^47^Sc, enabling multiple separations of in-grown ^47^Sc in the generator-like system. Using this approach, Domnanich et al. (Domnanich et al. 2017a) demonstrated that up to 2 GBq ^47^Sc can be produced by thermal neutron irradiation of enriched ^46^Ca targets. The optimized chemical isolation of ^47^Sc from the target material allowed the formulation of up to 1.5 GBq ^47^Sc with high radionuclidic purity (> 99.99%) in a small volume (∼700 μL), which was useful for labeling purposes. Three consecutive separations within 1 week were possible by isolating the in-grown ^47^Sc (Domnanich et al. 2017a; Pawlak et al. 2019).

#### Production of ^47^Sc using protons

Scandium-47 can also be produced using high energy proton reaction on ^48^Ti (Srivastava 2013; Srivastava and Dadachova 2001). The nuclear reaction on ^48^Ti was quite popular as the natural abundance is quite high (73.72%) and the low cross section of the ^48^Ti(p,2p)^47^Sc nuclear reaction can be partly compensated by the use of thick targets. However, this method suffers from the co-production of ^46^Sc (T_1/2_ = 83.79 d) which emits high energy gamma rays abundantly. This impurity is a major concern in the production of ^47^Sc both for the risk of unnecessary radiation dose and the regulatory constraints when stored at the therapy wards (Jafari et al. 2019).

Alternative production routes have been explored to try to overcome this issue using ^48^Ca, ^nat^V, ^44^Ca targets or photonuclear reactions (Mausner and Srivastava 1993; Szkliniarz et al. 2016; Sitarz et al. 2018; Srivastava and Dadachova 2001). The cyclotron production of ^47^Sc via the ^48^Ca(p,2n)^47^Sc nuclear reaction with a proton energy range of 24 → 17 led to a radionuclidic purity of only around 87%, due to ^48^Sc co-production (Misiak et al. 2017). Using enriched ^48^Ca for irradiation with 20 MeV protons may be a feasible route for the production of GBq activity levels of ^47^Sc, however, the prohibitively high cost of enriched ^48^Ca has made it impossible to implement this production route to date (Misiak et al. 2017). Still, the production of ^47^Sc in medical cyclotrons providing proton beams at energy range of 15–20 MeV via the ^48^Ca(p,2n)^47^Sc nuclear reaction on ^48^Ca enriched calcium oxide target could potentially provide wide access to this radionuclide (Braccini 2016). However, the co-produced ^48^Sc undermines the ^47^Sc purity, and its content strongly depends on the energy of protons impinging the target and on the thickness of the target material, feasibility of this approach has been studied in detail (Carzaniga and Braccini 2019). Another production route proposes the use of natural vanadium targets, since natural vanadium consists of two isotopes: for more than 99.75% it is formed by stable ^51^V while the very long-lived ^50^V (*T*_1/2_ = 1.4 × 10^17^ y) occurs only in 0.25%. Experimental data on ^nat^V can hence be interpreted in broad energy range as coming from a monoisotopic ^51^V target and reaction cross-sections can be derived. The cross-sections of the ^nat^V(p,x)^47^Sc nuclear reaction were measured up to 70 MeV proton beam (Jafari et al. 2019; Pupillo et al. 2019; Ditroi et al. 2016), the low reaction yields are the disadvantage of this approach.

#### Production of ^47^Sc using alpha beams

The α-particle irradiation of ^44^Ca targets at a cyclotron, inducing the ^44^Ca(α,p)^47^Sc nuclear reaction has been considered, though with low yield and radionuclidic purity. The advantage of the ^44^Ca(α,p)^47^Sc reaction lies in the short range of α projectiles in Ca target, allowing the use of a relatively small amount of ^44^Ca target material for small scale studies with ^47^Sc. For example, it is reported that 200 mg of [^44^Ca]CaO prepared in a diameter of 10 mm would give a yield of approximately 11 MBq at 10 eμA for 2 h irradiation at the end of preparation (approximately 1.5 h from the EOB) in the energy range of 28 → 0 MeV (Minegishi et al. 2016).

#### Production of ^47^Sc in photonuclear reactions

Photonuclear reactions in electron linear accelerators (linacs) using titanium (Jafari et al. 2019; Yagi and Kondo 1977; Mamtimin et al. 2015; Rotsch et al. 2018) and calcium targets (Starovoitova et al. 2015; Rane et al. 2015) have been explored. However, the cross sections are very small. It may be more convenient to use neutrons and ^46^Ca. Mamtimin et al. (Mamtimin et al. 2015) studied the production of ^47^Sc via the ^48^Ti(γ,p)^47^Sc nuclear reaction by Monte Carlo simulations. Rotsch et al. (Rotsch et al. 2018) evaluated the production yields and purification of the photonuclear-produced ^47^Sc from natural titanium oxide targets. In the recent report on photonuclear production Loveless et al. (Loveless et al. 2019a) used eLINAC to produce ^47^Sc via ^48^Ti(γ,p)^47^Sc reaction. They irradiated a stack of natural titanium foils using bremsstrahlung radiation generated by impinging 22 MeV electrons onto a 0.762 mm thick tungsten radiator. Despite the long irradiation times (10.5–14 h) low activity (approx. 2 MBq) was produced with 90% ^47^Sc, 1.2% ^46^Sc and 8.3% ^48^Sc at EOB. The authors suggested the use of enriched titanium, available in oxide form for low cost. It is expected to improve the attainable radionuclidic purity, but not significantly increase the yield, as the natural abundance of ^48^Ti is 73.7%. Importantly, the use of oxide target material requires a special target design, to effectively dissipate the heat during irradiation. Accelerator-based photoproduction of ^47^Sc in ^48^Ca(γ,n)^47^Ca → ^47^Sc nuclear reaction was also reported by Starovoitova et al. (Starovoitova et al. 2015) and Rane et al. (Rane et al. 2015), the latter aiming to develop the ^47^Ca/^47^Sc generator obtained from irradiated ^48^Ca target.

To date there is no preferred ^47^Sc production route. The presence of radionuclide contaminants is expected in all cases. The choice depends on the availability of the irradiation sites and enriched target materials. For example, higher ^46^Ca enrichment may favour its neutron irradiation. At the sites operating linear electron accelerators the photonulear reactions will be preferred (Qaim 2019).

### Targetry

Calcium enriched in ^44^Ca, required to reach high radionuclidic purity of ^44^Sc, is available only in salt form (oxide, carbonate). The low heat conductivity of the target limits the beam current during irradiation. At high beam currents, the heat accumulation caused burnout and/or cracking of the irradiated target and may lead to gas production through the thermal dissociation of CaCO_3_ (Wojdowska 2019). In some application, the issue of low thermal conductivity was handled by pressing the calcium carbonate on top of graphite powder to facilitate heat transfer and to hold the calcium carbonate powder in position (van der Meulen et al. 2015). The improvment of the heat conducitivity is paid by a lower production as part of the projectile will interact with carbon atoms. However, this method yielded up to 2 GBq of ^44^Sc when using proton beam energies of near 11 MeV, but can not be automatized easily (see Table 2). Calcium oxide obtianed through thermal decomposition of calcium carbonate, both natural and enriched in ^44^Ca, was used for preparation of disk shaped pellets which were encapsulated into aluminum for irradiation. Thus increased density of calcium oxide improved the irradiation yield compared to carbonate, though due to the higroscopicity of CaO to the pellets needed to be protected from moisture during storage and when exposed to irradiation (van der Meulen et al. 2020).

Alternatively, magnesium or aluminium powder were used as an additive for target pellet preparation (Mikolajczak et al. 2018; Stolarz et al. 2018). Nuclear reactions on these atoms under 16 MeV proton energy lead to short lived isotopes mainly, resulting in relatively little radioactive contaminants at the end of irradiation. The magnesium pellet can be selectively dissolved from an aluminium target holder with 3 M hydrochloric acid. Aluminum is also easily dissolved after removal from the holder coin.

**Fig. 1:**
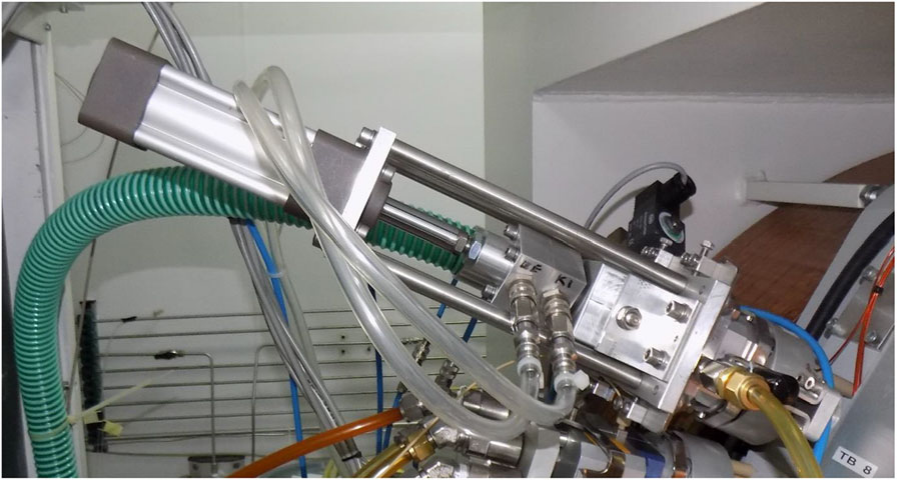
Solid target system with pneumatic target transfer, developed at the University of Debrecen

Introduction of the powdery target material (calcium oxide or calcium carbonate) directly into the cyclotron represents a high risk of contamination as the material may evaporate during irradiation. If a metal foil is used as a target material cover to prevent evaporation, the choice of metal needs careful consideration as it will also act as a degrader. When the foil is too thin, it may separate from the target material during irradiation and may burn out at higher beam currents, as it was reported at 27 μA 16 MeV on a 12.5 μm aluminum foil by Severin et al. (Severin et al. 2012). Conversely, if a higher degrader thickness is required to adjust optimal energy on the target, it has to be actively cooled (van der Meulen et al. 2015). In medical cyclotrons it is safer to use helium-cooled HAVAR foils to separate the irradiated target material from the vacuum system of the cyclotron (e.g., the ARTMS system). In the target system developed in Debrecen (Mikolajczak et al. 2018), the original parts from a water target were used as an interface to the beam port of the cyclotron (see Fig. 1). This enabled the circulation of helium cooling gas between the two foils. After several months of regular use, the contamination caused by calcium evaporation was clearly visible on the internal surfaces of the target system on the second foil. However, no carbonate migrated through the foils to the cooling cycle, or to the high vacuum side.

The high investment costs and the complicated installation are usually mentioned as major drawbacks of a solid target system. Due to the more complex operation, solid target handling systems will be always more expensive than a simple liquid target but will produce 10 times more radioactivity. Installation of a shuttle type solid target system (commercially available from several companies) requires 4–5 cm diameter passage in the walls of the cyclotron bunker and the hot lab, and some free space in the floor duct between them. However, this can be simply made at one end and nearly impossible at another in already existing facilites.

The challenges related to solid target systems prompted the investigation of ^44^Sc production in a liquid target. Hoehr et al. (Hoehr et al. 2014) irradiated approximately 1.5 g/mL natural calcium nitrate solution with 13 MeV proton beam in a relatively small volume liquid target. 28 MBq ^44^Sc was produced with a 20 μA beam current for 1 h, which can reach a gigabecquerel level, if enriched target material is used. However, it is questionable, whether the production can be managed in a cost-efficient way given that a high amount of enriched material has to be irradiated (approx. 100 times more, than for a solid target production from ^44^CaCO_3_). The use of liquid targets for radiometal production offers easier installation and lower hardware investment costs, but the presence of corrosive liquids on a cyclotron, which is producing ^18^F for daily FDG production is of high concern. IBA (Nirta Ga liquid) and GE (gallium target) also offer liquid targets for radiometal production, however, evidence showing their long-term use is needed. From the regulatory point of view, it might be necessary that a liquid target is used for the production of only one radiometal. In contrast, a solid target system can be used to irradiate targets for several radiometals using dedicated target holders.

Gelbart et al. (Gelbart and Johnson 2019) developed a hybrid target system to overcome the installation problems of shuttle type systems. The equipment, used for target dissolution is located in the cyclotron bunker behind the irradiated target, allowing the transport of the produced radioisotope in solution to the hot lab. It was designed for ^99m^Tc and ^68^Ga production, but can be utilized for scandium as well.

### Chemical processing

Several separation methods have been described in the literature, each strongly related to the form of the initial target material. Those proposed for separation of scandium comprised mostly solvent extraction (Zhang et al. 1997; Vibhute and Khopkar 1985; Rane and Bhatki 1966; Radhakrishnan and Owens 1972; Kalyanaraman and Khopkar 2002). However, for the radioactive isotopes of scandium extraction chromatography is preferred. Cartridge- or small column-based extraction chromatography methods can be automatized easily, and enable reproducible purification. Considering the further development of scandium radionuclides production, the technical issues associated with irradiation, target handling, dissolution and processing need to be solved. For routine application in clinical trials and future diagnostic use, these steps should be automated in order to facilitate the production and to meet the requirements of GMP and radiation protection. Pourmand et al. (Pourmand and Dauphas 2010) reported the strong Sc(III) retention on DGA resin and the negligible Ti(III) retention at HCl molarities below 6 M. After elution with 4.0 mL of HCl 0.1 M scandium is concentrated in a smaller volume by the use of second column containing SCX cation exchange resin and eluted with 4.8 M NaCl/0.13 M HCl effluent. The same approach was used by van der Meulen et al. at PSI, Villigen (van der Meulen et al. 2015). A summary of different approaches developed in the literature is given in Table 3.

**Table 3:** Separation methods related to the initial target material for getting scandium radionuclides

Starting material	Separation Method	Reference
**TiO**_**2**_	ion exchange the AG MP-500 (Bio-Rad) was used after a preliminary treatment in 5 M HNO_3_ and rinsing with H_2_O. Ti(IV) was poorly sorbed. Subsequently, scandium was eluted using ammonium acetate	(Pietrelli et al. 1992)
	solvent extraction tri-n-butylphosphate (TBP) was used with an equal volume of 8 N HCl, washed with H_2_O and a 6% of carbonate solution. Equal volumes of aqueous phase of ion solution and TBP were used. Cupferron (ammonium salt of the conjugate base derived from N-nitroso-N-phenylhydroxylamine) was also used and Ti(IV) was extracted as cupferrate by 100% chloroform, Sc was separated from Ti by “gravity” with 98% of Sc extracted	(Pietrelli et al. 1992; Valdovinos et al. 2015
	extraction chromatography tri-n-butylphosphate (TBP) was used with an equal volume of 8 N HCl, washed with H_2_O and a 6% of carbonate solution. Equal volumes of the aqueous phase of ion solution and TBP were used. TBP sorbed onto silica showed that 97.7% of Sc(III) were eluted with 2 mL of HCl 0.1 N. No titanium was detected in the final samples	(Pietrelli et al. 1992)
	extraction chromatography DGA resin could be used for Ti/Sc trace separations in the context of a fine purification of ^44^Ti from the residual scandium target material. By contrast, ZR® resin was shown to exhibit a high sorption affinity for titanium, whereas scandium could be eluted with HCl solutions. Nonetheless, there are some drawbacks concerning this generator since some breakthrough of ^44^Ti has been observed after several bed elutions.	(Majkowska-Pilip and Bilewicz 2011)
**Ti(0)**	extraction chromatography Dissolution in NH_4_HF_2_ Elution on a branched DGA resin, recovery of scandium 88%	(Polosak et al. 2013; Loveless et al. 2019b)
**CaCO**_**3**_	extraction chromatography target dissolved in HCl solution, passed through a UTEVA® resin column and the column washed with HCl. The scandium radionuclides were eluted with H_2_O. Efficiency 80%	(Valdovinos et al. 2015; Muller et al. 2014)
	extraction chromatography dissolving the CaCO_3_ targets in HCl solution, passed through a DGA® resin with HCl. Afterwards, the acidic ^47^Sc solution was passed throught SCX cation exchange cartridges cation and eluted with HCl. Efficiency 93%	(Domnanich et al. 2017a)
	extraction chromatography dissolving the CaCO_3_ targets in HCl solution, passed through a DGA® resin with HCl. Afterwards, the acidic ^43^Sc or ^44^Sc solution was loaded on a column filled with DOWEX50 cation exchange resin and ^43^Sc or ^44^Sc was eluted using ammonium acetate solution at pH = 4. Efficiency 75%	(van der Meulen et al. 2015; Muller et al. 2014; van der Meulen et al. 2020)
	Ion exchange dissolution of the target in HCl and adsorption of ^43^Sc or ^44^Sc and even ^47^Sc onto a chelating ion exchange resin Chelex 100. After adsorption of Sc, the column was washed with 0.01 M HCl to remove Ca^2+^, scandium was eluted with 1 M HCl. Efficiency 70%	(Walczak et al. 2015; Krajewski et al. 2013; Gizawy et al. 2020)
	extraction chromatography loading the dissolved target acidic solution onto DGA®; rinsed in 4 M HCl; HCl was then necessary to elute quantitatively scandium from the DGA® resin. Efficiency 95%	(Chaple and Lapi 2018; Alliot et al., 2015a; Filosofov et al. 2010)
	extraction chromatography dissolution of the target in 9 M HCl, loaded on TBP resin column, ^47^Sc eluted with H_2_O. Than loaded onto a BioRad AG50WX4 resin, washed with HCl 0.1 M and H_2_O; ^47^Sc was eluted with portions of 1 M sodium acetate solution at pH 4.5. Separation yield 52–79%	(Rotsch et al. 2018)
	extraction chromatography target dissolved in 11 M HCl solution, passed through a UTEVA® resin column. Scandium radionuclides were eluted with H_2_O and loaded on a BioRad AG50WX4 resin, washed with HCl 0.1 M and ^44^Sc was eluted with portions of 1 M sodium acetate solution at pH 4.5 Separation yield 93.5%	(Muller et al. 2018)
	extraction chromatography dissolution of the target in 1 mL 2 M HCl, partial neutralization with 0.7 mL 1 M NaOH, pH adjustment with pH 3 formate buffer loading on Nobias PA-1 (iminobisacetic acid–ethylenediaminetriacetic acid chelate resin) resin column, washing with 2 mL formate buffer, pH 3, elution with 0.1 mL 2 M HCl, separation yield: 95%	(Kilian et al. 2018)
	Precipitation calcium target dissolved with 2 M HCl, pH adjusted to 6.5–9.0 by addition of 1 M NH_4_OH, solution pushed through a 0.22 μm Millex-GV 13 mm diameter syringe filter washed with 10 mL 0.1 M NH_4_OH adjusted to pH 8–9 with HCl.	(Severin et al. 2012)
	extraction chromatography target dissolved in HCl solution, ^44^Sc was separated from excess of calcium by precipitation of scandium hydroxide using ammonia. passed through a UTEVA® resin column and the column washed with HCl. The scandium radionuclides were eluted with H_2_O. Efficiency 80%	(Wojdowska 2019)
	electroamalgamation selective electroamalgamation of Ca^2+^ ions	(Chakravarty et al. 2017)
	precipitation Dissolution of the target in 1 M HCl, then alkalized with 25% ammonia. Method takes advantage of the insolubility of Sc(OH)_3_ either as a precipitate or coprecipitate, which can be separated from calcium by using microfilters with PTFE membrane (0.22 μm)	(Minegishi et al. 2016; Severin et al. 2012; Duval and Kurbatov 1953)

The purity of the resulting batches is of great importance. Most of the published works dealt with ^44^Sc. These studies have shown that a high chemical purity of the final ^44^Sc fraction is important, since the presence of other metals may interact with the DOTA-chelator (or any other chelator) and, hence, the radiolabeling yield would be compromised. The concentration of common environmental contaminants (Al(III), Cu(II), Pb(II), Zn(II), Fe(III)) in ^44^Sc is frequent. The most problematic is Fe^3+^ for which the stability with the DOTA ligand is greater than that for Sc^3+^. By contrast, the influence of divalent metal cations (considered as contaminants) is negligible due to the much lower stability of their DOTA complexes. In order to meet the requirements for radiopharmaceutical applications, the obtained final solution containing scandium radionuclide needs to be of high chemical purity and concentrated into a small volume of moderately acidic eluate to facilitate efficient radiolabeling and subsequent in vivo application. Most of the times, authors do not indicate the final chemical purity of the resulting bacthes.

### Target recovery

Calcium has six naturally occurring isotopes (^40^Ca, ^42^Ca, ^43^Ca, ^44^Ca, ^46^Ca, and ^48^Ca), where ^40^Ca is the most abundant, comprising about 97% of naturally occurring calcium. However, calcium doesn’t form gaseous compounds near room temperature and atmospheric pressure, hence commonly used enrichment schemes such as gas centrifuge or thermal diffusion are not strictly impossible, but challenging. On the other hand methods based on liquid calcium forms, such as ion exchange chromatography or electrophoresis are difficult to scale up. Hence, enriched calcium materials are very expensive and a recycling process of the target needs to be developed (Jalilian et al., 2020). The choice of method used in the recovery process is related to the form of the initial target material, and it is particularly important when large amounts of the highly enriched target material are used. The recycling will contribute to a significant cost reduction, considering that the target material alone costs around 20–25 € per 1 mge (mg element) of [^44^Ca]CaCO_3_ dependig on the purchased quantity (price for 2020). Additionally, the purchased material needs to be purified before use so the method for recycling applies here as well.

The published data on this topic is quite limited. Few publications deal with the calcium carbonate targets (natural or enriched in ^44^Ca) for production of ^44^Sc in cyclotrons. One of the early works by Krajewski et al. (Krajewski et al. 2013) reported the use of chelating resin Chelex 100 for separation of ^44^Sc and target recycling efficiency of only 60%. In the work by Alliot et al. (Alliot et al. 2015a), using the mixture bicarbonate/methanol, the rate of solvent evaporation was increased and the solubility of calcium carbonate was lowered (Kan et al. 2002). The recovery yield of enriched calcium of 90 ± 2% was obtained when starting with CaCO_3_ material and the HCl solutions from the extraction process. The solution was then loaded on a pre-conditioned AG1 × 8 column to retain all metallic impurities (Cu, Co, Fe…) and enriched ^44^Ca was rinsed in 9 M HCl. This solution was evaporated to dryness and the dry residue recovered in a mixture of bicarbonate buffer (pH = 10.33)/methanol, filtered and the residue dried in an oven at 105 °C to remove water and methanol. The recycled targets were irradiated, and no significant difference in production yield was observed (Alliot et al. 2015a). The improved recovery reaching 98% of the initial enriched material, significantly reducing the operational costs, was reported by Van der Muelen et al. (van der Meulen et al. 2015) who collected effluents from the DGA column and recycled enriched calcium through simple and fast precipitation as Ca-oxalate, which was then converted to carbonate by slow heating to 500 °C. Alternatively, the final step involved heating at 900 °C to ensure conversion to CaO. It was shown that recycled material could be used effectively for ^44^Sc production (van der Meulen et al. 2020). In the production of ^47^Sc with alpha beams Minegishi et al. (Minegishi et al. 2016) recycled enriched target, collected as ^44^Ca^2+^ in the waste fraction via the precipitation with ammonium carbonate and further dehydration to ^44^CaO by decomposition of ^44^CaCO_3_ at 940 °C for 2 h. The recovery rate of approximately 85–90% (by weight), with negligible loss.

When starting material is TiO_2_, the enriched titatnium may be recovered and reused. A simple procedure was developed that recovers around 98.5% of the oxide based on precipitation of titanium at basic pH followed by conversion of the oxide using higher temperature (Kolsky et al. 1998). Other processes for recovery of titanium from its acidic solutions use either HF and HNO_3_ or converting to nano-sized titanium dioxide (Kolsky et al. 1998). The use of TiO_2_ as a starting material and its recycling process have been reported by Loveless et al. (Loveless et al. 2019b). The HCl solution and HNO_3_ obtained from the extraction processes were collected and NH_4_OH at pH 8 was added to precipitate TiO_2_. The precipitate was then heated for 4 h at 400 °C. Optionally, the enriched titanium oxide can be reduced with calcium or calcium hydride, resulting in titanium pellets with low content of impurities (Lommel et al. 2013).

### Chelators for Sc

Scandium with its ionic radius (r_i_) 74.5 pm (CN = 6) is chemically similar to Y^3+^ and the heaviest lanthanides. Similarly to them, scandium is almost exclusively present in its compounds in the trivalent state. Therefore, ligands developed for these cations should be also suitable for chelating Sc. However, the chemistry of trivalent scandium has some differences compared to lanthanides; it is smaller (thus, harder and has a higher preference for hard oxygen donor ligands), and prefers donor numbers from six to eight (Kerdjoudj et al. 2016).

The multi-dentate ligands, which were already used in Gd(III)-based MRI contrast agents as well as for radiolanthanides, that is derivatives of DTPA or DOTA, were also the first choice for scandium chelation for medical applications (Mausner et al. 1995). The stability constant of Sc-DOTA complex was comparable to that for Lu^3+^ and heavier lanthanides but higher than those for In^3+^ and Ga^3+^. The ^13^C NMR studies have shown that Sc(DOTA) similarly to Lu(DOTA) forms in solution complexes with eight-coordination geometry (Majkowska-Pilip and Bilewicz 2011)^.^ The stability constants of scandium(III) complexes DTPA and DOTA (log *K*_ScL_ 27.43 and 30.79 respectively) were determined from potentiometric and ^45^Sc NMR spectroscopic data. Both complexes were fully formed even below pH 2. Complexation of DOTA with the Sc^3+^ ion was much faster than with trivalent lanthanides. Proton-assisted decomplexation of the [Sc(dota)]^−^ complex (*τ*_1/2_ = 45 h; 1 M aq. HCl, 25 °C) was much slower than that for [Ln(dota)]^−^ complexes (Pniok et al. 2014). Therefore, DOTA and its derivatives were assumed to be very suitable ligands for scandium radioisotopes (Pniok et al. 2014; Huclier-Markai et al. 2015).

Thermodynamic data for scandium(III) complexes with polyamino-polycarboxylic ligands, such as NOTA, EDTA or TETA have been determined using potentiometric titration and free ion selective radiotracer extraction (FISRE) method and the values of stability constants were found to be in the order TETA<NOTA<EDTA<DTPA<DOTA. (Huclier-Markai et al. 2011) DOTA derivatives with phosphinic/methylphosphonic acid pendant arms (i.e. DO_3_A; DO_3_AP^PrA^, DO_3_AP^ABn^) were also investigated showing that the stability constants of the monophosphinate analogues were somewhat lower than that of the Sc(DOTA) complex. (Kerdjoudj et al. 2016) The thermodynamic stability constant of recently developed chelating agent AAZTA, Sc(AAZTA) was reported to be lower than that of Sc(DOTA) but the striking difference was observed on the radiochemical yield at 25 °C indicating that AAZTA quickly incorporated ^44^Sc (Nagy et al. 2017a). The AAZTA derivative AAZTA^5^ (1,4-bis (carboxymethyl)-6-[bis (carboxymethyl)]amino-6-[pentanoic-acid]perhydro-1.4-diazepine) was synthesized representing a bifunctional version with a pentanoic acid at the carbon-6 atom. [^44^Sc]Sc-AAZTA^5^ complexes as well as [^44^Sc]Sc-AAZTA^5^-TOC were formed at room temperature within 5 min in the pH range 4 to 5.5 and were very stable (Sinnes et al. 2019). Another chelator, H_4_pypa (N_5_O_4_) has been shown to exhibit high complexation constant with Sc (log K = 27) as well as with In, Lu, Y and La, as determined by potentiometric titration and UV spectrometry (Li et al. 2019a; Li et al. 2019b). Its radiolabeling could be performed at room temperature and in a quite wide range of pH values within 10 min. Also, it has been conjugated to a Glu-urea-Lys based PSMA (prostate-specific membrane antigen, e.g. PSMA-617). When labelled with ^44^Sc (pH = 4.5, 30 min RT) this conjugate showed specific tumor uptake (4.86% ID/g from ex vivo biodistribution, 8 h p.i.) without significant off-target uptake, except in the kidney. (Li et al. 2019b) Further search on chelators enabling Sc chelation at room temperature led to the development of the small-cavity triaza-macrocycle-based, picolinate-functionalized chelator H_3_mpatcn. Spectroscopic and radiochemical studies established the [Sc(mpatcn)] complex as kinetically inert and appropriate for biological applications. As a proof-of-concept bifunctional conjugate targeting the prostate-specific membrane antigen (PSMA), picaga-DUPA, chelated ^44^Sc to form {^44^Sc}Sc(picaga)-DUPA at room temperature with an apparent molar activity of 60 MBq μmol^− 1^ and formation of inert *RRR*-Λ and *SSS*-Δ-twist isomers. Sc(picaga)-DUPA exhibited a *K*_i_ of 1.6 nM for PSMA and the ^44^Sc labelled Sc(picaga)-DUPA revealed high-quality images in prostate cancer-bearing animals. H_3_mpatcn and its bifunctional analogue picaga represent new additions to the chelator toolbox for the emerging ^44/47^Sc theragnostic isotope pair as reported by Vaughn et al. (Vaughn et al. 2020a). Nonetheless, the molar activity calculated from their experimental data was estimated to be 0.03 MBq/nmol and thus the metal to ligand ratio was ranging between 1: 485,500 to 1: 1,084,600. Hence, picagaDUPA seems exhibiting complexation/radiolabeling at RT but complexation constant must be established and the potential of this ligand to provide higher molar activity must be evaluated with sources of ^44^Sc of high purity.

Scandium also displays favorable physical properties and chemistry for conjugation to mAb-chelate systems. Since it is chemically similar to ^90^Y and close to ^177^Lu, the same ligands developed for ^90^Y or ^177^Lu can be used for chelating ^47^Sc.

Table 4 provides a summary of the main chelates that have been evaluated, with the corresponding formula and the measured value of the complexation constant with scandium. The table reports values reported in the original manuscripts, accuracy of which is not homogeneous across the data. For simplicity, the uncertainties (available only for selected studies) are not reported.

**Table 4:** Selected chelators for scandium and their complexation constants

Acronym of the chelate	Name in the IUPAC nomenclature	Scheme of the molecule	Complexation constant with scandium Only log K_**ScL**_	Reference
DOTA	1,4,7,10-tetraazacyclododecane-1,4,7,10-tetraacetic acid		30.79	(Pniok et al. 2014)
AAZTA	1,4-bis(carboxymethyl)-6-[bis(carboxymethyl)]amino-6-methylperhydro-1,4-diazepine		27.69	(Nagy et al. 2017a)
NOTA	1,4,7-triaazacyclodeodecane-1,4,7-tetraacetic acid		18.5	(Huclier-Markai et al. 2011)
EDTA	Ethylene diamine tetra acetic acid		23.1	Huclier-Markai et al., 2011
DTPA	Diethylene Triamine Pentaacetic Acid		27.43	(Pniok et al. 2014)
TETA	Tri-EthyleneTetra-Amine		18.0	Huclier-Markai et al., 2011
DO3AP	1,4,7,10-TetraazacycloDOdecane-1,4,7-Tri-Acetic-10-methylPhosphonic acid		27.75	(Kerdjoudj et al. 2016)
DO3AP^PrA^	10-{[(2-carboxyethyl)hydroxyPhosphoryl]methyl}-1,4,7,10-tetraazacycloDOdecane-1,4,7-Tri-Acetic acid		25.75	(Kerdjoudj et al. 2016)
DO3AP^ABn^	1,4,7,10-TetraazacycloDOdecane-4,7,10-Tri-Acetic-1-{methyl[(4-aminophenyl)methyl]Phosphinic acid}		25.51	(Kerdjoudj et al. 2016)
H4pypa	pyridinecarboxylate- based ligand		27	(Li et al. 2019b)
mpatcn	1,4-bis(methoxycarbonyl)-7-[(6-carboxypyridin-2-yl)methyl]-1,4,7-triazacyclononane		Constant not determined	(Vaughn et al. 2020a; Vaughn et al. 2020b)
picagaDUPA	prostate-specific membrane antigen (PSMA) - 2-[3-(1,3-dicarboxypropyl)ureido]pentanedioic acid (DUPA)			(Vaughn et al. 2020a; Vaughn et al., 2020b)
3,4,3 HOPO	hydroxypyridinone (HOPO)		25.16	(Carter et al. 2020)

### Radiolabeling studies

Labeling protocols should allow high labeling yields, radiochemical purity and molar activity (Fani and Maecke 2012). Labelling efficiency of ligands is usually tested at various pH, temperature and ligand concentration (or more correctly, at different radiometal-to-ligand molar ratios) and is monitored as a function of time in order to optimize the radiolabeling. Herein, we discuss the development of labelling methods that were developed for scandium radionuclides. Neverthelles, the reported data are often difficult to compare. To overcome this problem, the recommendations on consensus nomenclature rules for radiopharmaceutical chemistry should be followed (Coenen et al. 2017).

Most of the published radiolabeling works have been done with ^44^Sc. All have highlighted the importance of reaching a high chemical purity of the final ^44^Sc fraction before radiolabeling. The presence of other metals may interact with the DOTA-chelator (or any other chelator) that can affect the radiolabeling yield. The content of environmental contaminants (i.e. Al(III), Cu(II), Pb(II), Zn(II), Fe(III)) in ^44^Sc is frequent. The most problematic is Fe^3+^, for which the stability with the DOTA ligand is greater than that for Sc^3+^. By contrast, the influence of divalent metal cations (considered as contaminants) is negligible due to the lower stability of DOTA-divalent metal complexes. Although DOTA forms very stable complexes, it exhibits slow formation kinetics at room temperature, that could be increased by heating. However, elevated temperatures remain an important obstacle for efficient labelling of heat-sensitive molecules such as antibodies. Click-chemistry or the new ligands permitting the formation of scandium complexes with faster kinetics, or at much lower temperatures than that required for DOTA, might circumvent this issue. In this context, AAZTA or H4pypa seem to be interesting alternatives (Nagy et al. 2017a; Li et al. 2019a), although their availability is limited.

Table 5 provides a summary of the radiolabeling studies published to date. Notably, there are many discrepancies in presentation of results and in most of the cases the quality control results of the batches are not reported. When available, radiochemical yield and molar activity are indicated. This inhomogeneity of the published data makes the direct comparison of radiotracers efficacy difficult. The data in Table 5 are reported as published in the original papers, pointing to the discrepancies in the measurement units used. Indications on the quality control procedure are also given as well as the conditions of post-radiolabeling purification, wherever reported. The use of suitable buffers, stabilizers/free radical scavengers etc. was addressed.

**Table 5:** Radiolabeling studies performed on scandium radionuclides

Type of vector	RN used	Buffer, pH, T°, time conditions	Quality Control / RCY	Molar activity	Post-radiolabeling purification	Reference
DOTA	^44^Sc	0.2 M ammonium acetate pH = 4.8 80 °C 30 min	iTLC with H_2_O	51 GBq/μmol competitive metal impurities of 28 μM (mostly Fe)	Not indicated	(Severin et al. 2012)
DTPA HEBD BAPTA EGTA TTHA DFO DOTA	^47^Sc	20 mM acetate buffer pH = 6.0 RT	iTLC with NH_3_/H_2_O (1/25) DTPA RCY = 38.8–100% EGTA RCY = 97.2–100% DOTA RCY = 79.4–97.7%	Estimation 0.017 MBq/nmol	Not performed	(Severin et al. 2012; Polosak et al. 2013)
picagaDUPA	^44^Sc	0.25 M NaOAc buffer solution pH = 4 at 25 or 80 °C.	TLC with 50 mM EDTA RCY = 83% at 25 °C RCY = 96% at 80 °C	Calculated from (Polosak et al. 2013) 0.03 MBq/nmol	Not performed	(Vaughn et al. 2020a)
AAZTA-c(RGDfK)	^44^Sc	0.25 M ammonium acetate pH = 4 room temperature 5 min	RCY = 99%	0.36 GBq/μmol		(Nagy et al. 2017a; Vaughn et al. 2020a; Kostelnik and Orvig 2019)
Propargyl-DOTA	^44^Sc	95 °C for 30 min	HPLC Phenomenex Synergi, 4 μ, Hydro-RP, 80 Å a gradient with the eluents acetonitrile (A) and H_2_O (B), both containing 0.1% trifluoroacetic acid and a flow rate of 1.0 mL/min. RCY = 69–92%	Other metals: Zn (5.4 ± 4.5 ppm), Fe (1.1 ± 0.7 ppm), Pb (0.38 ± 0.13 ppm) and Al (0.18 ± 0.14 ppm)	Not performed	(Hoehr et al. 2014)
DOTA - puromycin	^44^Sc	0.25 M ammonium acetate pH =4 95 °C 20 min	iTLC Elution with n-propanol/ NH_4_OH/H_2_O (55:35:10) RCY = 78%	1.5 GBq/μmol	HPLC - C-18 cartridge (Strata-X Polymeric Sorbet 60 mg/mL Phenomenex) Elution with ethanol Radiochemical purity ≥97%	(Eigner et al. 2013)
DOTA-Folate	^44^Sc	1 M ammonium acetate pH 3.5–4 95 °C 10 min	HPLC - C-18 column (Xterra MS C18, Waters). Mobile phase: Milli Q water with 0.1% trifluoracetic acid (A) and methanol (B) with a linear gradient from 95% A and 5% B to 20% A and 80% B over 25 min with a flow rate of 1 mL/min RCY ≥ 96%	≥ 5.2 MBq/nmol, representing a ^44^Sc–to–ligand molar ratio of 1:5600	Not indicated	(Muller et al. 2018)
	^47^Sc	CH_3_COONH_4_/HCl pH 4.5 95 °C 10 min	HPLC as above RCY ≥ 96%	no radionuclidic impurities were detectable 13 MBq/nmol	Not indicated	(Muller et al. 2014)
PSMA-617	^44^Sc	0.25 M ammonium acetate pH = 4 95 °C 25 min	iTLC 1:1 v/v 1 M ammonium acetate / methanol RCY = 74%- 98%	1.82–6.69 MBq/nmol	HPLC - C-18 cartridge (Sep-Pak C18 Plus Short Waters) - after a pre-conditioning with 4 mL of ethanol and 10 mL of water. Elution with pure ethanol.	(Eppard 2018)
DOTANOC	^44^Sc	0.2 M sodium acetate buffer pH 4 85 °C 15 min	HPLC C-18 (XterraTM MS column 95% MilliQ water with 0.1% trifluoracetic acid (A) and 5% acetonitrile (B) over a 20-min period at a flow rate of 1 mL/min RCY ≥ 98%		Not indicated	(van der Meulen et al. 2015)
	^47^Sc	0.5 M sodium acetate pH 4.5 95 °C 15 min	HPLC C-18 (XterraTM MS column 95% MilliQ water with 0.1% trifluoracetic acid (A) and 5% acetonitrile (B) over a 20-min period at a flow rate of 1 mL/min RCY ≥ 96%	10–25 MBq/nmol	Not indicated	(Domnanich et al. 2017a)
DOTATOC	^44^Sc	0.8 M ammonium acetate pH = 5 91 °C 30 min	iTLC RCY = 99%	1.4 MBq/μg	Not indicated	(Singh et al. 2017)
	^44^Sc	0.25 M ammonium acetate pH = 4 95 °C 30 min	iTLC a) 0.1 M sodium citrate pH 4.0 (b) mixture of 5% NaCl with MeOH (3:1) (c) mixture of 5% NaCl with MeOH and 25% NH_3_ (3:1:1) RCY ≥ 98%	9 GBq/μmol	Performed on RP C-18 mini-cartridge Strata-X Elution with pure ethanol	(Kerdjoudj et al. 2016)
DOTATATE	^44m/44^Sc	0.1 M ammonium acetate pH 4–6 70 °C 20 min	TLC 25% aq. NH_3_/H_2_O/MeOH, 2/1/1 (v/v) RCY ≥ 95%	8 MBq/nmol radionuclidic purity = 99%	Not performed	(Huclier-Markai et al. 2018; Alliot et al. 2015a; Alliot et al. 2015b)
	^43^Sc	ammonium acetate pH = 5 90 °C 35 min	TLC 0.1 M sodium citrate (pH = 5.0) RCY = 98.3%	Not indicated	Not performed	(Szkliniarz et al. 2016)
CHX-A”-DTPA-Fab fragment of Cetuximab	^44^Sc	0.5 M sodium acetate buffer pH 6.5 adjusted to ∼4.5 room temperature (25 °C) 30 min	TLC 50% aqueous acetonitrile	63 GBq/μmol	PD-10 columns PBS as the mobile phase	(Chakravarty et al. 2014)
DOTA -Z_HER2–2891_	^44^Sc		RCY = 98%	7.8GBq/μmol		(Honarvar et al. 2017)

### Pre-clinical studies

The use of radiolabeled compounds for in vitro and in vivo preclinical animal studies has steadily increased in recent years, becoming a much more widely used method for studying biochemistry, physiology and pharmacology (Kilbourn and Scott 2018). The increasing availability of ^44^Sc and its compatibility with numerous chelators, among those DOTA, has led to multiple studies in animals, especially imaging studies. The half-lives of ^43^Sc, ^44^Sc or ^47^Sc are compatible with the pharmacokinetics of a fairly wide range of targeting vectors. Several somatostatin analogs, either DOTA-derivatized (DOTATOC, DOTATATE, DOTANOC) or with NODAGA as a chelator, have been labeled with scandium radionuclides to prove the suitability of the obtained radionuclide solution for radiolabeling (Pawlak et al. 2019; Loveless et al. 2019a) or to compare under the same conditions the in vitro/in vivo imaging/therapeutic potential of scandium radiolabeled peptides to their ^68^Ga- or ^177^Lu (^90^Y) – radiolabeled counterparts (Walczak et al. 2015; Loveless et al. 2019a; Singh et al. 2017; Pruszynski et al. 2012). The scandium radionuclides labeled bombesin analogs (Koumarianou et al. 2012), RGD peptides (Domnanich et al. 2017c), folate derivatives (Muller et al. 2018; Muller et al. 2014; Siwowska et al. 2019) and PSMA ligands (Umbricht et al. 2017) have been evaluated pre-clinically. Few studies have also reported on scandium radiolabeled antibodies, antibody fragments or affibodies (Chakravarty et al. 2014; Honarvar et al. 2017; Moghaddam-Banaem 2012) and nanoparticles (Eppard et al. 2018). A summary is given in Table 6.

**Table 6:** Current state of pre-clinical studies performed on scandium radionuclides

Type of vector	Radioisotope used	Tumor model or human	Bio-Distributions (BioD) PET images	Main results	Reference
DOTATATE DOTANOC DOTATOC	^nat^Sc, ^43^Sc, ^44^Sc	AR42J cells, xxx	In vitro		(Walczak et al. 2015; Minegishi et al. 2016; Domnanich et al. 2017b; Pruszynski et al. 2012; Domnanich et al. 2017c; Koumarianou et al. 2011)
DOTATOC	^47^Sc	AR42J cells	In vitro		(Loveless et al. 2019a)
DOTA - puromycin	^44^Sc	Walker carcinoma 256 (breast carcinoma) in rats AT1 carcinoma (prostate carcinoma) in rats	BioD + PET	significant tumor uptake of [^44^Sc]Sc-DOTA puromycin and a clear-cut tumor visualization, cellular uptake of [^44^Sc]Sc-DOTA-puromycin could be suppressed by blocking protein synthesis	(Eigner et al. 2013)
DOTA-Bombesin	^44^Sc, ^68^Ga	PC3 cells, rats with Dunning R-3327-AT-1 prostate cancer tumor (GRP/BN receptor expression) MCF7	BioD + microPET	^68^Ga- and [^44^Sc]Sc-DOTA-BN[2–14]NH_2_ showed no differences in tumor uptake in micro-PET images [^68^Ga]Ga- and [^44g^Sc]Sc-DOTA-Ava-BBN showed similar accumulation and retention profiles in PC3 and MCF7 tumors	(Koumarianou et al. 2012), (Ferguson et al. 2020)
DOTA-Folate	^44^Sc	KB cells KB tumor-bearing mice	BioD + PET	Tissue accumulation is similar to [^177^Lu]Lu-folate = > ^44^Sc could serve for dosimetry prior to ^177^Lu-based therapy	(Muller et al. 2018; Muller et al. 2014)
	^47^Sc	KB cells KB tumor-bearing mice	BioD	Delay in tumor growth	(Muller et al. 2014; Siwowska et al. 2019)
PSMA-617	^44^Sc, ^177^Lu, ^68^Ga	PC-3 PIP and PSMA-negative PC-3 flu prostate cancer cells	BioD + PET (2 h p.i.)	[^44^Sc]Sc-PSMA-617 almost identical biodistribution data compared to [^177^Lu]Lu-PSMA-617	(Umbricht et al. 2017)
	^43^Sc, ^47^Sc, ^44^Sc	LNCaP cells + human	BioD + PET + SPECT (2 h p.i.)	Tumor to liver ratio between 2.5 and 8.8.	(Umbricht et al. 2017; Eppard et al. 2017)
picagaDUPA	^44^Sc	PC3 cells, Male NCr nude mice	BioD + PET (90 min p.i.)	uptake 13.8 ± 0.6% ID/g enhanced binding affinity to the target for [^44^Sc]Sc(picaga)-DUPA, and possibly also from slower blood clearance.	(Vaughn et al. 2020a; Vaughn et al. 2020b)
DOTA-RGD NODAGA-RGD DOTA-NOC NODAGA-NOC	^44^Sc, ^68^Ga	U87MG (human glioblatoma) and AR42J tumor-bearing mice	BioD + PET/CT (0.5 to 5 h p.i.)	^44^Sc stability using NODA-functionalized peptides was increased compared to the corresponding radiolabeled one with ^68^Ga	(Domnanich et al. 2017c; Hernandez et al. 2014)
DOTA-NAPamide	^44^Sc, ^68^Ga	MC1-R positive (B16-F10) and negative (A375) melanoma cell lines.	In cellulo, BioD + PET/CT (1 to 4 h p.i.)	Uptake 2.61 ± 0.46% ID/g on B16-F10 and 0.21 ± 0.08% ID/g on A375 for ^44^Sc-DOTA-NAPamide	(Nagy et al. 2017b)
CHX-A”-DTPA-Fab fragment of Cetuximab	^44^Sc	U87MG (human glioblastoma) in tumor- bearing mice)	PET + BioD (0.5 to 6 h p.i.)	rapid tumor uptake (max. Uptake of ∼12% ID/g at 4 h p.i.) of with excellent tumor-to-background ratio	(Chakravarty et al. 2014)
DOTA-Z_HER2–2891_	^44^Sc	HER2-SKOV3.ip (human ovarian cancer) in vitro and in xenografted mice	BioD (6 h p.i.)	Higher tumor-to-background contrast	(Honarvar et al. 2017)
DOTA-antiCD20	^46^Sc	Raji cells in wild rats	BioD (72 h p.i.)	similar biodistribution pattern compared to other radiolabeled anti-CD20 immunoconjugates	(Moghaddam-Banaem 2012)

The early study by Koumarianou et al. (Koumarianou et al. 2011) was conducted on “cold” complexes with natural scandium and gallium. The binding affinity of both ^nat^Sc-DOTATATE and ^nat^Ga-DOTATATE in AR42J cell line was in the sub-nanomolar range, but in favor of ^nat^Ga-DOTATATE. The affinity to GRP (gastrin releasing peptide) receptors in the PC-3 cell line was higher for ^nat^Ga-DOTA-BN[2–14]NH_2_ than that of ^nat^Sc-DOTA-BN[2–14]NH_2_ (IC_50_(nM) values 0.85 ± 0.06 vs. 6.49 ± 0.13). Despite this difference, in another study both ^68^Ga- and ^44^Sc-labeled DOTA-BN[2–14]NH_2_ showed comparable biodistribution and micro-PET imaging. (Koumarianou et al. 2012). Similar observations were reported by Ferguson et al. who compared [^44g^Sc]Sc-DOTA-Ava-BBN, a bombesin receptor antagonist, to the [^68^Ga]Ga-DOTA-Ava-BBN in breast and prostate cancer model (Ferguson et al. 2020). In more recent studies, the binding affinity of ^nat^Sc-PSMA-617 was evaluated in comparison to ^nat^Ga-PSMA-617, however using different methods and cell lines (PC-3 PIP cells and PSMA+ LNCaP cells, respectively) (Umbricht et al. 2017; Eppard et al. 2017). Though, the binding affinity to the target was found to be in the same molar range. Both [^44^Sc]Sc- and [^68^Ga]Ga-PSMA-617 exhibited similar in vivo behavior, with [^44^Sc]Sc-PSMA-617 displaying higher tumor-to-liver ratios at 15 and 30 min p.i. These values correlated more closely to [^177^Lu]Lu-PSMA-617 than to [^68^Ga]Ga-PSMA-617, and therefore were considered useful for pre-therapeutic dosimetry (Umbricht et al. 2017).

The comparison of ^44^Sc and ^68^Ga for imaging of animals with melanocortin-1 receptor (MC1-R), positive tumors using DOTA-NAPamide, at 4 h p.i. revealed significantly higher tumor uptake of ^44^Sc (81.7 ± 7.7%ID/g) over ^68^Ga (17.3 ± 1.85%ID/g) (Nagy et al. 2017b). DOTA-puromycin was radiolabeled with the generator produced ^44^Sc and investigated for the potential imaging of protein synthesis in vivo*.* In μPET images of tumor-bearing rats significant tumor uptake of [^44^Sc]Sc-DOTA-puromycin and a clear-cut tumor visualization were demonstrated. In addition, the cellular uptake of [^44^Sc]Sc-DOTA-puromycin could be suppressed by blocking protein synthesis (Eigner et al. 2013).

In vitro, [^47^Sc]Sc-folate demonstrated effective reduction of folate receptor-positive ovarian tumor cell viability similar to ^177^Lu-folate, but ^90^Y-folate was more potent at equal activities due to the higher energy of emitted β^−^-particles. Comparable tumor growth inhibition was observed in mice that obtained the same estimated absorbed tumor dose (~ 21 Gy) when treated with [^47^Sc]Sc-folate (12.5 MBq), [^177^Lu]Lu-folate (10 MBq), and [^90^Y]Y-folate (5 MBq), respectively. However, there were no statistically significant differences among the therapeutic effects observed in treated groups (Siwowska et al. 2019).

CHX-A”-DTPA has been successfully used for radiolabeling of the monoclonal antibody (mAb) Cetuximab. (Chakravarty et al. 2014) Another study evaluated DOTA-HPMA (N-(2-hydroxypropyl)methacrylamide) conjugates for labeling efficiency with ^68^Ga, ^177^Lu and ^44^Sc and showed that the ^44^Sc labeled polymer allowed for in vivo PET imaging and ex vivo measurements of organ distribution for up to 24 h (Eppard et al. 2018).

### Clinical experiences

The validity, usefulness and advantages of ^44^Sc have been demonstrated by studies featuring ^44^Sc-radiolabeled targeting vectors, including ^44^Sc radiopharmaceuticals in early clinical studies. The first of them has been performed using [^44^Sc]Sc-DOTATOC prepared from ^44^Sc produced in a cyclotron (Singh et al. 2017). Two patients were included in this study after being treated by peptide receptor radionuclide therapy due to neuroendocrine neoplasms. The obtained image quality was comparable to that of ^68^Ga. This very encouraging proof-of-concept study showed no clinical adverse effects with normal hematology, nor with renal and hepatic profiles.

The other studies were conducted in patients with metastasized castrate-resistant prostate cancer (Eppard et al. 2017; Khawar et al. 2018a; Khawar et al. 2018b). A first-in-human investigation was carried out in a cohort of four patients (mean age 70.0 ± 1.8 years) registered for [^177^Lu]Lu-PSMA-617 therapy. Physiological tracer uptake was observed in kidneys, liver, spleen, small intestine, urinary bladder, and salivary glands and pathological uptake in both soft and skeletal metastases. SUV values were significantly lower in the kidneys (14.0) compared to [^68^Ga]Ga-PSMA-11 PET (30.5). All other measured SUV values did not show a statistically significant difference. Tumor-to-liver ratios were found to lie between 1.9 and 8.3 for [^68^Ga]Ga-PSMA-11 and between 2.5 and 8.8 for [^44^Sc]Sc-PSMA-617 after 120 min. For [^44^Sc]Sc-PSMA-617 the ratios were higher and no statistically significant differences were observed. Total and % activity were highest in the liver followed by kidneys, spleen, small intestine and salivary glands. Rapid wash-out was seen in liver and spleen, and gradually over time in kidneys. Kidneys received the highest radiation absorbed dose of 0.354 (0.180–0.488) mSv/MBq. No adverse pharmacological effects were observed. Still, the authors concluded that the clinical advantages for individual dosimetry or other applications like intraoperative applications need to be investigated in further studies (Eppard et al. 2017).

The impact of physical properties of ^44g^Sc on image quality has been studied by Bunka et al. (Bunka et al. 2016) and recently evaluated by Rosar et al. (Rosar et al. 2020) in comparison to ^68^Ga with different imaging phantoms. The lower mean positron energy of ^44g^Sc (0.63 MeV) compared to ^68^Ga (0.83 MeV) can result in better spatial image resolutions. However, high-energy γ-rays (1157 keV) are emitted at high rates (99.9%) during ^44g^Sc decay, which can reduce image quality. Despite the presence of high-energy γ-rays in ^44g^Sc decay, a higher image resolution of small structures was observed with ^44g^Sc when compared to ^68^Ga. Structures as small as 1 mm could be visualized and analyzed using two different pre-clinical PET scanners. Recently, Lima et al. (Lima et al. 2020) evaluated quantitative capabilities of ^44^Sc-PET using a commercial PET scanner and concluded that that clinical ^44^Sc-PET imaging has the potential to provide signal recovery in lesions of different sizes comparable to current ^18^F-PET standards.

Biodistribution and radiation exposure to normal organs with [^44^Sc]Sc-PSMA-617 in metastatic castration-resistant prostate carcinoma patients were investigated by Khawar et al. (Khawar et al. 2018a). These authors extrapolated the pharmacokinetics of [^44^Sc]Sc-PSMA-617 to that of [^177^Lu]Lu-PSMA-617 and demonstrated that pharmacokinetics of [^44^Sc]Sc-PSMA-617 PET/CT imaging could be utilized with the intent of predicting normal organ-absorbed doses and maximum permissible activity in patients scheduled for therapy with [^177^Lu]Lu-PSMA-617. Physiological tracer uptake was seen in kidneys, liver, spleen, small intestine, urinary bladder, and salivary glands and metastases. Kidneys, with the highest radiation absorbed dose of 0.319 mSv/MBqm were the critical organs, followed by the urinary bladder wall, spleen, salivary glands, and liver. Red marrow dose was found to be 0.0331 mSv/MBq. The mean effective dose of 0.0389 mSv/MBq and effective dose of 1.95 mSv was estimated from 50 MBq (treatment planning dose) of [^44^Sc]Sc-PSMA-617 (Khawar et al. 2018a). These first clinical studies using [^44^Sc]Sc-DOTA-TOC and [^44^Sc]Sc-PSMA-617 confirmed the potential of ^44^Sc-labeled tracers as promising radiopharmaceuticals, their use is especially relevant in pre-therapeutic dosimetry (Kostelnik and Orvig 2019).

To date, very limited data is available on radiation doses from ^47^Sc labelled radiopharmaceuticals. The absorbed dose in human organs of ^47^Sc-EDTMP (ethylene-diamine-tetramethylene-phosphonic acid) has been extrapolated from animal biodistribution using the MIRDOSE formalism. However the animal biodistribution data in this study were collected using EDTMP radiolabeled with the reactor produced ^46^Sc of rather low specific activity 148 MBq/mg (Deilami-Nezhad et al. 2017). The radionuclide ^47^Sc is a low energy beta emitter, similarly to ^177^Lu, and the mean range of its radiation in bone is 0.20 mm, compared to 0.15 mm for ^177^Lu (as extrapolated from Bouchet et al. (Bouchet et al. 2000)).

### Quality specifications

The chemically identical radiopharmaceuticals for diagnosis and therapy using the matched pair ^43^Sc or ^44^Sc with ^47^Sc are very appealing. Although several groups are now producing scandium radionuclides locally and a few centers demonstrated the potential of Sc radionuclides for medical applications, the benefit of their use in the clinical setting is yet to be shown. Given the amount of work and documentation needed to obtain the approval for a clinical trial of a new radiopharmaceutical, this review aims to present the production options for Sc radionuclides and related quality constraints. This information might be useful when implementing Sc radionuclides production technology in the existing or planned production facilities.

^43^Sc (β^+^ = 88.1%, E_β+_ = 476 keV) and^ 44g^Sc (β^+^ = 94.3%, E_β+_ = 632 keV) have half-lives of 3.89 h and 3.97 h, respectively (Singh and Chen 2015). Although properties of these two isotopes are very similar, the main difference is the high-energy γ-radiation (Eγ = 1157 keV; Iγ = 100%) associated with ^44g^Sc. The high energy gamma radiation, similarly to that of ^89^Zr (Eγ909 keV; Iγ = 99%), may not hamper its clinical use. It is considerd advantageous for ^44g^Sc as the radionuclide of choice for 3-γ photons imaging, a new modality of imaging (Sitarz 2020). Additionally, ^44m^Sc, which is co-produced with ^44g^Sc, has a half-life of 58.6 h and has been suggested for use as an in vivo PET generator for ^44g^Sc (Alliot et al. 2015a). However, the longer half-life coupled with the high-energy γ-ray emitted from ^44g^Sc may lead to the unfavorable dosimetry for shorter biodistribution studies (Alliot et al., 2015a).

High radionuclidic purity ^44g^Sc production with proton irradiation necessitates the use of enriched target material and a recycling procedure to keep the cost reasonable. The determination of acceptable limits for each radionuclidic impurity requires careful consideration, as it can have an adverse effect on the implementation of new production methods. Potential contamination with ^43^Sc can be argued to have no impact on patient safety and quality of images since it is a positron emitter with no high energy gamma radiation component, in contrast to the expected radiation burden due to ^44g^Sc contamination in ^43^Sc. ^44m^Sc decays to ^44^Sc with low energy gamma (271 keV) emission, which does not cause any harm to the patient. The limit for ^44m^Sc content should be determined to avoid unnecessary radiation doses to the patient, because of the longer effective half-life of the ^44m^Sc/^44^Sc isotope pair. However, the biological half-life and critical organs should be determined for unchelated scandium isotopes before using worst-case dosimetry calculations for the determination of the limit of longer half-life impurities, as discussed earlier for ^68^Ga labelled peptides and influence of ^68^Ge breakthrough on dosimetry (Velikyan et al. 2013).

From that perspective, ^43^Sc has the most favorable radiation characteristics for conventional PET, however it can be produced efficiently only using alpha particle beams. ^44^Sc is in the most advanced state and has the highest potential for broad applications. Medical cyclotrons that currently supply ^18^F to hospitals can be used to produce ^44g^Sc or ^43^Sc, with a solid target system. The use of liquid target might not be reasonable as the amount of enriched material nedded is high. With the near to 4 h half-life and reasonable production cross-section, there is a potential for regional distribution following mass production at a single cyclotron unit with solid target. With the increasing numbers of installed cyclotrons, the availability of ^43/44^Sc may increase. Aiming for the production of high activities, a solid target is needed. This requires the addition of specialized hardware on the cyclotron and in the processing hot-cell, which is available from cyclotron providers but not frequently installed on ^18^F production machines. The co-emission of a high-energy γ-ray similar to ^89^Zr has to be taken into consideration when planning for radiation protection. If not controlled, it may increase the radiation dose to the patient and staff. ^44^Ti/^44^Sc generator currently has a high production cost and requires a regular and efficient use of the generator over long periods. ^44m^Sc half-life enables transportation of ^44m^Sc-labeled radiopharmaceuticals to hospitals that are located quite far away from the radiopharmaceutical production site (centralized production). This is based on the in-vivo generator principle.

Both positron emitters, ^43^Sc and ^44g^Sc can be used for theranostic studies with ^177^Lu or other lanthanides. Scandium has chemistry close to ^177^Lu and possibility of centralized production at the regional level, which makes it more desirable than ^68^Ga (local production only) for its use in a theranostic pair. Application of ^47^Sc, as an alternative radionuclide to ^177^Lu, was proposed in earlier works (Srivastava 2013). The advantage of ^47^Sc production compared to that of n.c.a. ^177^Lu, is the relatively easy isolation of the radionuclide from the target, but the disadvantage is the smaller cross-section of the nuclear reaction compared to ^177^Lu production. Nonetheless, the main concern lies with the co-produced impurities. Another limitation concerns the availability of the starting enriched material needed for the set-up of efficient separation chemistry. At the time of writing this article, we are aware of several research groups developing ways of overcoming these limitations, as presented by Jalilian et al. (Jalilian et al. 2020).

When it comes to the scandium-based radiopharmaceuticals, unlike with ^68^Ga, the scientific community involved is unfortunately far from following a standardized procedure. From Table 5, it is inferred that neither the metallic impurity contents nor the radionuclidic purity of the resulting radiolabeled compound is always given. There is also a lack of homogeneity in the expression of the molar activity of the resulting radiolabeled vector. Though, some compromise could be found on the radiolabeling protocol and quality control. This is certainly a good basis to pave the way for a monograph for the European Pharmacopeia, together with the use of the recently approved nomenclature guidelines for radiopharmaceuticals (Coenen et al. 2017).

Next to the radionuclide generators and kits, the European Union (EU) directive 2001/83 (The European Parliament and the Council of European Union 2001) dealing with radiopharmaceuticals, defines also the “radionuclide precursors” (EU Commission 2004). The radionuclide precursor is defined as “any other radionuclide produced for the radio-labeling of another substance prior to administration” and as such has to hold a marketing authorization when introduced to the market. Even if used in a hospital radiopharmacy with a cyclotron on-site, and having a status of starting material, as postulated in a position paper by Neels et al. (Neels et al. 2019), the radionuclide precursor needs to be controlled by performing several quality control tests (identity, purity, assay, etc.). To assure safe use in humans, these tests and limits should comply with certain quality standards, and be supported by a suitable quality management system. It is generally accepted that such quality standards are established in Pharmacopoeia monographs.

The expected final form of the radionuclide, regardless of the production method used, is the form of solution for radiolabeling. The Ph.Eur. General monograph on Radiopharmaceutical preparations (0125) can serve as the reference for establishing quality specifications for the final product solution of ^43^Sc, ^44^Sc or ^47^Sc (EDQM 2020a). One should bear in mind that the Ph.Eur. monographs on Lutetium (^177^Lu) solution for radiolabelling (mon. 2798) (EDQM 2020b), and Yttrium (^90^Y) chloride solution for radiolabelling (mon. 2803) (EDQM 2020c), can be also very informative, similarly as the monograph of Gallium-68 (^68^Ga) chloride solution for radiolabelling (mon. 2464) (EDQM 2020d).

One needs to remember, however, that the specified parameters will depend on the production route (target material, nuclear reaction, accompanying nuclear reactions, chemical processing etc.). For example, when using ^44^Sc obtained from the ^44^Ti/^44^Sc radionuclide generator, the potential breakthrough of ^44^Ti will be of concern as well as its impact on the quality (and safety) of the final radiopharmaceutical. The detailed specifications for quality control of generator eluate as well as for the quality assessement of ^44^Sc radiolabeled PSMA-617 prior to its administration to patients were developed by Eppard (Eppard 2018).

The Group 14 (radioactive compounds) of European Pharmacopoeia elaborated the *Guide for the elaboration of monographs on radiopharmaceutical preparations*, European Pharmacopoeia, EDQM Edition 2018, which should help to prepare the monographs for new radiopharmaceuticals (https://www.edqm.eu/sites/default/files/guide_-_guide_for_the_elaboration_of_monographs_on_radio-pharmaceutical_preparations_-_october_2018.pdf). Based on this Ph. Eur. Guide, exemplary general list of tested parameters and the involved methods for the potential scandium radionuclide precursors are briefly discussed below.

### Title, definition and production sections

In general, the definition states that the monograph applies to the substance obtained by a certain route of production and in the case of a radionuclide precursor the name of the substance is completed by “for radiolabeling”. Therefore, in case of the new monographs, e.g. for gallium-68 and technetium-99 m, the title of the monograph itself contains a reference to the production method, like: *Gallium (*^*68*^*Ga) Chloride (Accelerator-Produced) Solution For Radiolabelling (mon. 3109)* (EDQM 2020e) or *Sodium Pertechnetate (*^*99m*^*Tc) Injection (Accelerator-Produced (mon. 2891)* (EDQM 2020f)*.*

Such titles and definitions are intended to make the recipient/reader aware that other methods of obtaining radionuclides may and, in fact do, result in a completely different profile of potential contaminants, mainly radionuclidic. These constraints apply to the radionuclides of scandium, therefore it is not possible to develop a single monograph even for one of the scandium radionuclides, but it would be necessary to develop several monographs for this radionuclide depending on the way the radionuclide is produced. In the following, a monograph-like characteristics are proposed.

#### Characters

Appearance: clear, colourless solution.

Half-life and nature of radiation of scandium radionuclides:

Scandium-47: T_1/2_ = 3.35 d, Eβ^−^av = 162 keV, Eγ = 0.159 MeV.

Scandium-43: T_1/2_ = 3.89 h, 88% Eβ^+^, Eβ^+^av. = 476 keV, Eγ = 0.373 MeV (23%).

Scandium-44: T_1/2_ = 4.04 h, 94.27% Eβ^+^_and 5.73% EC, Eβ^+^av. = 632 keV, Eγ = 1.157 MeV (99.9%).

#### Identification: (characteristic of type of radiation - spectra, approximate half-life, pH, chromatography)

Gamma-ray spectrometry.

The most prominent gamma photon of scandium-47 has an energy of 0.159 MeV.

The most prominent gamma photon of scandium-43 has an energy of 0.373 MeV.

The most prominent gamma photon of scandium-44 has an energy of 1.157 MeV.

pH: 1.0 to 2.0, using a pH indicator strip R.

#### Tests

##### Radionuclidic purity

For ^90^Y, ^177^Lu and ^68^Ga (generator produced) the Ph.Eur. recommended limit is minimum of 99.9% of the total radioactivity, however, for the accelerator-produced ^68^Ga (draft monograph 3109 published in Pharmeuropa 30.4) the radionuclidic purity limit is only 98% due to the expected and the inevitable presence of contaminants such as ^66^Ga and ^67^Ga.

The expected radionuclide contaminants in ^47^Sc radionuclide precursor are ^46^Sc (T_1/2_ = 83.8 d) and ^48^Sc (T_1/2_ = 43.7 h). Although their presence has been reported, the limits for individual radionuclidic impurities were not yet determined.

In case of ^43^Sc, irradiation of ^46^Ti targets (97.0% enriched) with protons yielded a product of high radionuclidic purity, containing 98.2% ^43^Sc and only 1.5% ^44^Sc. Long-term γ-spectroscopy measurements determined low activity levels of 0.079% ^44m^Sc, ^46^Sc, ^47^Sc, ^48^Sc and 0.34% ^44m^Sc, ^47^Sc, ^48^Sc in the final products of irradiated ^46^Ti and ^43^Ca targets, respectively. (Domnanich et al. 2017b).

##### Chemical purity

The ions of copper, iron, zinc and lead and the traces of target material such as Ca or Ti.

Since DOTA is the most common chelator used for labeling with ^47^Sc, it can be assumed that the content of chemical impurities such as copper, iron, zinc and lead ions may have an impact on ^47^Sc quality. The content of chemical impurities may vary depending on the processing method and purity of reagents. The methods used for determination of chemical contaminants included: determination of Ti using ICP-MS or determination of metallic impurities by ICP atomic emission spectrometry. These instrumental techniques can be recommended to establish chemical impurity acceptance levels.

Although the influence of metallic impurities on the labelling yields of DOTA-chelated peptides has been studied in detail, the first report dealing with Sc labelled DOTATATE is just recently published by Walczak et al. (Walczak et al. 2019).

##### Radiochemical purity

In case of ^68^Ga and ^111^In, the Ph.Eur. monographs for diagnostic radionuclide precursors (2464, 1227) require the presence of gallium or indium min. 95% in + 3 ion form, whereas for therapeutic radionuclides like ^177^Lu (mon. 2798) and ^90^Y (mon. 2803) the limit is increased to min. 99% in + 3 ion form. It seems that these rules should be also adopted in case of ^43^Sc and ^44^Sc radionuclide precursors and ^47^Sc, respectively.

In the Ph.Eur. monographs for ^177^Lu and ^90^Y solutions for radiolabeling, for the assessment of radiochemical purity, in addition to the chemical determination of the radiometal form as trivalent cation, the TLC system recommends using complexes of these radiometals with pentetic acid as reference.

To test the radiochemical purity of obtained ^47^Sc when used for radiolabeling of selected common chelators such as DOTA, DTPA or EGTA, or peptide conjugates such as DOTATATE or DOTA-bombesin, with further TLC development to assess the radiochemical purity of ^47^Sc-labelled compound. Usually, the radiochemical purity should be not less than 98–99% to confirm the quality of ^47^Sc.

This approach can also serve as the indirect test of ^47^Sc suitability for radiolabeling, and the experiments can be designed to check the effective specific activity of obtained ^47^Sc solutions. Such tests provide very useful information during the process development, especially when instrumental methods for determination of chemical purity are not available on-site.

##### Bacterial endotoxins

General applicable limit for the presence of bacterial endotoxins in radiopharmaceutical preparations is less than 175 IU/V, V being the maximum volume to be used for the preparation of a single patient dose, if intended for use in the manufacture of parenteral preparations without a further appropriate procedure for the removal of bacterial endotoxins. Whereas, however, it should be borne in mind that the limit of 175 IU/V applies to the final injection preparation, therefore the determination of the limit of bacterial endotoxins for the radionuclide precursor should take into account the additional effect of the labelled substance and should therefore be established on the basis of actual results obtained for several batches of the radionuclide precursor intended for radiolabeling.

##### Sterility

The test applies if intended for use in the manufacture of parenteral preparations without a further appropriate sterilization procedure.

From the regulatory point of view, labeling efficiency of ligands is usually tested at different pH, temperature and ligand concentrations (or more precisely, at different radiometal-to-ligand molar ratios), and monitored as a function of time to optimize the radiolabeling.

The preparation of ^43/44/47^Sc-radiolabeled peptides for patient administration is designed to be performed on a modular system in which the final radiotracer is typically purified on a cartridge and diluted in a physiological vehicle before injection. Using the same commercially-available modular entity as is commonly used for the preparation of ^68^Ga-radiopharmaceuticals has the advantage to allow the preparation of ^43/44/47^Sc-radiopharmaceuticals for clinics without further evaluation and GMP validation of the system.

All these elements combined with a larger availability of the isotope production sites and broader distribution network are expected to allow more clinical trials with scandium radionuclides labelled radiopharmaceuticals (Domnanich et al. 2017c). These radionuclides have attracted considerable interest in the last decade and their broader availability opens new avenues for investigations.

## Conclusions

The ^44^Sc/^47^Sc or ^43^Sc/^47^Sc pairs are appealing true theranostic radionuclides for Nuclear Medicine. Reliable methodologies for the production of all discussed herein medical scandium radionuclides exist and are increasingly reliable. With the increasing availability of scandium PET isotopes, some preclinical studies have been conducted, but remain limited and mostly performed with peptides. Since scandium exhibits suitable conjugation chemistry to be coupled with MAbs, and ^44m^Sc half life is adapted to long biodistribution time, it paves the way for bringing new developments in this area. Scandium-based vectors from diagnosis to therapy offer a great opportunity for dosimetric calculations and the development of personalized medicine.

Among scandium radionuclides, the ^44^Sc seems to be in the most advanced state. Several production and purification methods were developed and some hold the promise of their relatively easy adaptation to the locally available infrastructure. At present, the primary importance of ^44^Sc lies in its potential broad-scale availability based on the production in medical cyclotrons and the possibility to use it as the diagnostic match with ^177^Lu or ^47^Sc. However, efficient production of ^47^Sc is still not developed though the half life of ^47^Sc allows its production in a centralized facility, which might provide this radionuclide to several countries. Its widespread use will be questionable if one considers the current availability of ^177^Lu and the problems associated with the use of a therapeutic agent without centralised marketing authorisation. The shorter half-life of ^44^Sc can be beneficial considering the lower radiation dose for the patients. The main issue is the co production of ^46^Sc when Ti target is used, but that is not a problem when produced through Ca target.

The ^44^Sc is an alternative to ^68^Ga, a well-established radiometal, with a similar field of application. The availability of ^68^Ga from generator gave a boost of its widespread use, but it is clear now, that after the initial success in the introduction of new diagnostics (somatostatin analogues and PSMA), production capacity and cost efficiency will be the main factors determining the future of these examinations, and the interest of centralized production. Additionally, the short half-life of ^68^Ga may limit its use for some pharmakokinetics studies. These factors favor fluorine labeling over radiometals. ^44^Sc can find its place in the palette of medical cyclotrons, which could be capable of producing scandium before the daily ^18^F irradiation. Using the [^18^F]FDG transportation channels for shipping ^44^Sc solution for radiolabeling or the GMP produced ^44^Sc tracers to the nuclear medicine departments facilitates the use of new specialized tracers, currently relying on the ^68^Ge/^68^Ga generator supply.

The authors trust that the medical applications of scandium radionuclides will be growing, however standardization of their quality is still needed. The question remains whether there is enough experience already to draft the pharmacopoeia monograph for any of scandium radionuclides as radipharmaceutical precursors and which of the scandium radionuclides becomes economically feasible.

## Data Availability

Data sharing is not applicable to this article as no datasets were generated or analysed during the current study.
